# The origin and evolution of queen and fertility signals in Corbiculate bees

**DOI:** 10.1186/s12862-015-0509-8

**Published:** 2015-11-16

**Authors:** Ricardo Caliari Oliveira, Cintia Akemi Oi, Mauricio Meirelles Castro do Nascimento, Ayrton Vollet-Neto, Denise Araujo Alves, Maria Claudia Campos, Fabio Nascimento, Tom Wenseleers

**Affiliations:** Department of Biology, Laboratory of Socioecology & Social Evolution, KU Leuven, Leuven, Belgium; Department of Biology, Laboratory of Behavioral Ecology, FFCLRP, University of São Paulo, Ribeirão Preto, Brazil; Department of Entomology and Acarology, ESALQ, University of São Paulo, Piracicaba, Brazil

**Keywords:** Fertility signals, Queen pheromones, Ancestral state reconstruction, Bees

## Abstract

**Background:**

In social Hymenoptera (ants, bees and wasps), various chemical compounds present on the cuticle have been shown to act as fertility signals. In addition, specific queen-characteristic hydrocarbons have been implicated as sterility-inducing queen signals in ants, wasps and bumblebees. In Corbiculate bees, however, the chemical nature of queen-characteristic and fertility-linked compounds appears to be more diverse than in ants and wasps. Moreover, it remains unknown how queen signals evolved across this group and how they might have been co-opted from fertility signals in solitary ancestors.

**Results:**

Here, we perform a phylogenetic analysis of fertility-linked compounds across 16 species of solitary and eusocial bee species, comprising both literature data as well as new primary data from a key solitary outgroup species, the oil-collecting bee *Centris analis*, and the highly eusocial stingless bee *Scaptotrigona depilis.* Our results demonstrate the presence of fertility-linked compounds belonging to 12 different chemical classes. In addition, we find that some classes of compounds (linear and branched alkanes, alkenes, esters and fatty acids) were already present as fertility-linked signals in the solitary ancestors of Corbiculate bees, while others appear to be specific to certain species.

**Conclusion:**

Overall, our results suggest that queen signals in Corbiculate bees are likely derived from ancestral fertility-linked compounds present in solitary bees that lacked reproductive castes. These original fertility-linked cues or signals could have been produced either as a by-product of ovarian activation or could have served other communicative purposes, such as in mate recognition or the regulation of egg-laying.

**Electronic supplementary material:**

The online version of this article (doi:10.1186/s12862-015-0509-8) contains supplementary material, which is available to authorized users.

## Background

In order to be able to effectively organize themselves, insect societies require reliable information and communication systems [1]. In social insects, various communication mechanisms have evolved, of which chemical cues and signals are likely the most important [2]. Chemical substances present on the cuticle of individuals can serve various purposes, from the primary function of preventing desiccation [3] to having roles in nestmate and species recognition [4–6], courtship regulation [7–9] and signalling caste or reproductive status [10–19]. With respect to the latter, it has recently been shown in some hymenopteran species that fertility-linked cuticular compounds help to regulate the reproductive division of labour between queens and workers in one of several distinct ways. For example, in the buff tail bumblebee, three ant species and the common wasp, it was shown that specific long-chain linear and methyl-branched alkanes act as sterility-inducing queen signals that stop the workers from reproducing [11, 12, 16, 19–23], reviewed in [24]. Moreover, in the common wasp, one of the main sterility-inducing pheromones was also shown to be used by the queen to mark her eggs, thereby enabling the workers to recognize and “police” eggs laid by other workers [25]. Finally, in two ants species, egg-laying workers were shown to produce specific alkenes that enable them to be recognized and policed via aggression by other workers [23, 26, 27].

The important role of cuticular hydrocarbons as queen pheromones in ants, wasps and the bumblebee contrasts with what is known from the honeybee, where most existing studies have indicated that mandibular gland compounds, including the keto acid 9-oxo-decenoic acid (9-ODA), act as the principal sterility-inducing queen signals [24]. Nevertheless, even there, it is likely that some cuticular fertility-linked compounds are also active as queen pheromones, since queens from which the mandibular gland was removed still inhibited worker reproduction [28, 29] and that extracts of the tergal glands, which produce specific compounds on the dorsal part of queen’s cuticle [30, 31], also partially inhibited worker ovary development. In addition, in the stingless bee *Friesella schrottkyi*, it has recently been shown that specific linear and methyl branched alkanes were characteristic for the queen and that non-polar cuticular queen extracts inhibited worker reproduction [32].

In order to gain more insight into the origin and evolution of social insect queen pheromones, several studies have started to apply formal phylogenetic methods, and these show that there is remarkably high evolutionary conservation of queen signals [12, 16, 33]. For example, an ancestral state reconstruction has shown that structurally related saturated hydrocarbons were the most common chemical class of fertility-linked cues across more than 60 species of ants, bees, and wasps [12]. In addition, identical or structurally related saturated hydrocarbons were found to be bio-active across different *Lasius* ants [16] and even across several independently evolved social insect lineages, such as ants, wasps and bumblebees [12]. These results were interpreted as implying that queen pheromones likely evolved from pre-existing fertility signals in solitary ancestors, in which they may have had a different function [12, 24]. Furthermore, it has been suggested that the presence of honest signals of fertility in primitively eusocial species could lead to conditional helping strategies, whereby the most fecund individuals receive most help, thereby facilitate the transition to advanced eusociality with a pronounced reproductive division of labour [34]. Yet despite the potentially huge importance of fertility signals in the evolution and maintenance of eusociality, many questions remain. For example, it remains unknown why in bees the chemical nature of queen-characteristic and fertility-linked compounds is much more diverse than in ants and wasps [12], how queen signals evolved across this group and how they might have been co-opted from fertility signals in solitary ancestors. In fact, in the current most extensive phylogenetic analysis of fertility signals across over 60 social insect species, bees were the least represented, with only eight species. Furthermore, the published analysis [12] did not comprise any solitary outgroups, thereby limiting the power to infer how queen pheromones could have evolved from ancestral fertility-linked signals in solitary ancestors.

In the present study, we test the hypothesis that queen signals evolved from fertility-linked cues present in solitary ancestors [24] and perform a phylogenetic analysis of such compounds across 16 species of solitary and eusocial bee species. We use data from literature but also include new primary data from a key solitary outgroup species, the oil-collecting bee *Centris analis*, which is the sister taxon of all Corbiculate bees [35, 36], as well as data from the highly eusocial stingless bee *Scaptotrigona depilis.* Including *Centris* as an outgroup in our analysis allowed us to study character states present just before the transition from a solitary to a social lifestyle. In this way, we are able to obtain important insight with respect to what ancestral signals queen pheromones could have evolved from, and provide important clues as to what could be bioactive queen pheromones across different groups of bees.

## Methods

### Data collection

Chemical data on the identity of fertility signals across different species of solitary and social bees were collected from the literature through a systematic review of published studies, in which we compiled chemical data from fourteen different species (Additional file 1: Table S3), as well as through inclusion of new primary data of two more species (see below). Chemical compounds were classified as fertility linked whenever they were overproduced by queens in comparison to workers in eusocial species, or mature females versus non-egg laying virgin females in solitary species. These differences in chemical profiles could be either quantitative or qualitative. In order to standardize our data as much as possible, we mainly focused on studies that analysed apolar whole-body extracts, which recovers primarily cuticular compounds, but also smaller amounts of gland-derived compounds, such as 9-ODA in the honeybee [37, 38]. In addition, we obtained new data on the identity of fertility-linked signals in a key solitary outgroup, the solitary Centridini bee *Centris analis*, which is the closest extant relative of the Corbiculate bees [35, 36], as well as data on the queen signals produced by the highly eusocial stingless bee *Scaptotrigona depilis*. The oil collecting bee *Centris analis* was sampled using trap-nests for solitary bees [39]. Egg laying females (*n* = 5) were sampled when they visited their nest cavities whereas virgin females (*n* = 8) were collected on the day they emerged from trap-nests that were placed in the laboratory. Egg-laying queens (*n* = 5) and workers (*n* = 15) of the stingless bee *Scaptotrigona depilis* were sampled from the experimental meliponary from the University of São Paulo. Cuticular chemical data for the two bee species were obtained through gas chromatography and mass spectrometry analysis. Detailed sampling methods and details on the chemical analyses are provided in supplementary material.

### Ancestral state reconstruction

Our total dataset comprised data on fertility-linked cuticular compounds across sixteen bee species, of which five were solitary and eleven eusocial (Additional file 1: Table S3). Compounds that were characteristic for either mature queens in social species or mature egg-laying females in solitary ones were grouped into different classes that correspond to known or presumed biosynthetic pathways (linear alkanes, branched alkanes, alkenes, alkadienes, aldehydes, alcohols, fatty acids, keto acids, esters, terpenes, terpene alcohols or lactones) [10, 40–44]. Subsequently, the presence or absence of at least one fertility-linked or queen characteristic compound belonging to one of each of these classes were coded as binary characters and ancestral states were reconstructed with Mesquite 3.01 [45] using maximum likelihood, based on the Markov *k*-state 1 parameter model, assuming equal rates of changes for both gains and losses. Phylogenetic relationships among species were based on the molecular phylogenies given in refs. [36, 46–48]. As branch lengths were not available in all cases, branch lengths were set equal to 1, which corresponded to assuming a punctuational mode of evolutionary change [49]. The likelihood threshold of 50% was used to consider that a given character state was likely present.

The advantage of our analysis being performed at the level of broad biosynthetic series is that in this way we were able to detect evolutionary conservation of fertility-linked compounds even if there would be small changes in the bioactivity of particular compounds across the study species. For example, overexpression of an elongase in queens of one particular lineage could result in an increase in chain length of fertility-linked linear or methyl-branched hydrocarbons [10, 50, 51] and would likely result in a change in the bioactivity of particular individuals compounds, but the use of this particular class of compounds as queen pheromones would still qualify as evolutionarily conserved. We should note, however, that the use of compounds belonging to a particular class as queen or fertility signals does not preclude other compounds of that same class also being used for other biological functions.

### Ethical note

Data collection with live animals during this experiment was carried out at the University of São Paulo within the framework of a research project approved by the federal government (CNPq-Brazil 402661/2012-5). According to the country’s law, research with bees is exempted from ethics committee approval when performed in research institutions.

## Results

**Fig. 1:**
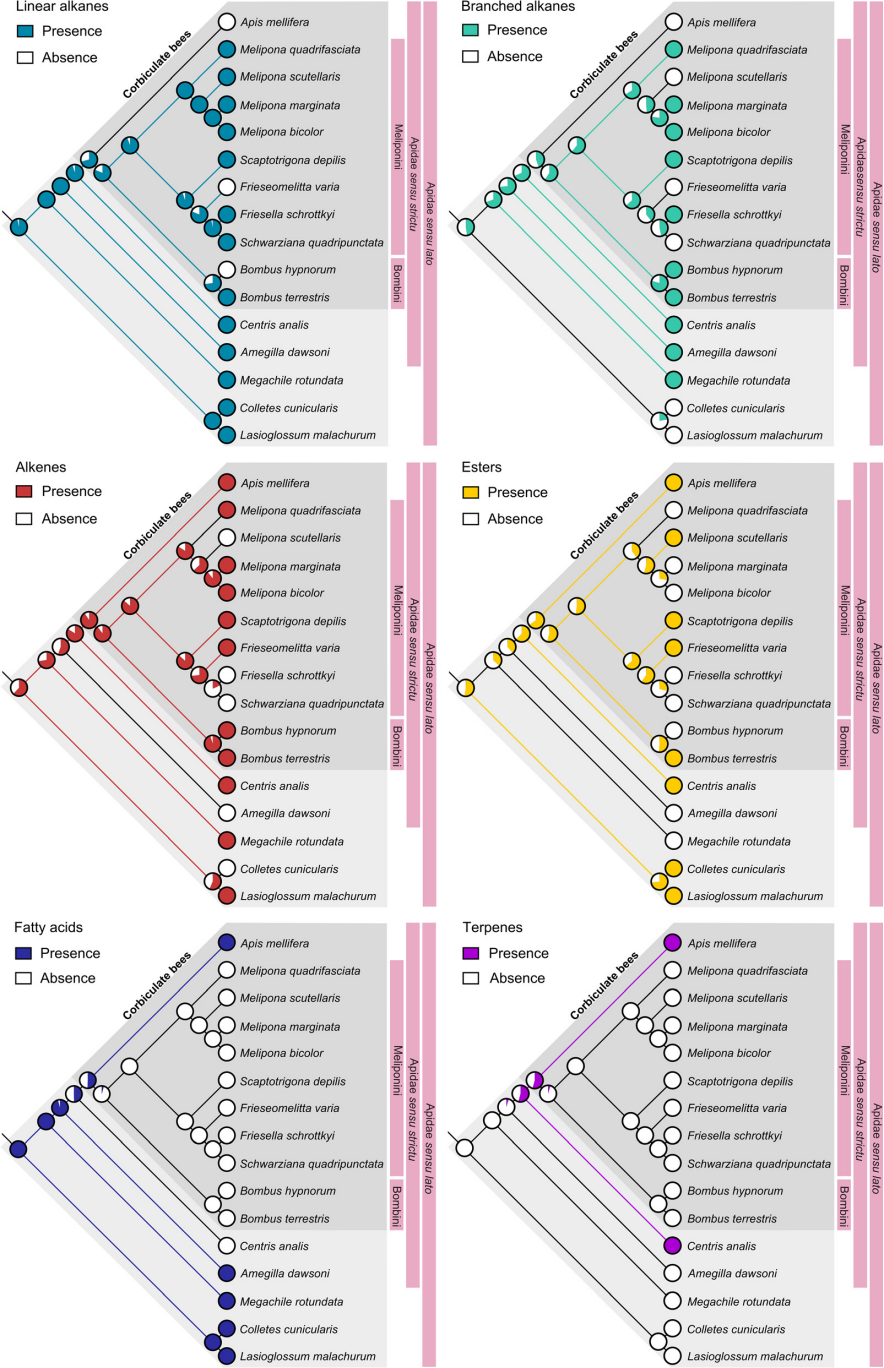
Maximum likelihood ancestral state reconstruction of six major classes of fertility signals in bees. Pies show the likelihood in percentage of a given character being present in the node and coloured branches represent branches for which the likelihood was greater than 50 %

Our results show that, in comparison to ants and wasps [12], there is a strikingly high diversity of fertility-linked chemical compounds in both solitary and eusocial bees. In particular, specific linear alkanes, alkenes, esters and fatty acids were already present with high likelihood (>50 %) as fertility-linked compounds in the ancestor of all solitary and social bees included in our analysis (Fig. 1, Table 1), whereas branched alkanes first appeared as fertility-linked compounds in the common ancestors of the Megachilidae and Apidae (Fig. 1, Table 1). In general, bees present odd long chain linear alkanes and alkenes ranging from heneicosane to hentriacontane, but other chain lengths including even chain length substances were also present in lower frequencies. Furthermore, specific terpenes also feature with high likelihood as fertility-linked signals in the common ancestor of *Centris* and the Corbiculate bees (Fig. 1, Table 1). Whereas both linear alkanes and alkenes are relatively conserved as fertility signals across all species analysed, fatty acids seem to have been lost in the common ancestor of stingless bees and bumblebees, and esters appear to have been gained and lost as fertility signals several times (Fig. 1). The use of keto acids, such as 9-ODA, as well as aldehydes, alcohols, alkadienes, terpene alcohols and lactones as queen or fertility signals appears to be restricted to specific lineages (Additional file 1: Figure S1).

**Table 1:** Maximum likelihood, in percentage, of a given fertility signal being present as ancestral state of different bee clades.

Compound Class	Apidae *sensu lato*	Megachile + Apidae *sensu strictu*	Apidae *sensu strictu*	*Centris* + Corbiculate bees	Corbiculate bees	Meliponini	Bombini
**Linear alkanes**	**96.9 %**	**99.0 %**	**98.8%**	**95.2 %**	**70.8 %**	**94.2 %**	**72.8 %**
**Branched alkanes**	47.5 %	**69.2 %**	**73.4 %**	**68.4 %**	44.3 %	**60.3 %**	**79.3 %**
**Alkenes**	**61.8 %**	**72.7 %**	**55.5 %**	**83.7 %**	**90.4 %**	**86.2 %**	**94.7 %**
**Esters**	**52.4 %**	36.5 %	38.3 %	**62.2 %**	**65.0 %**	**51.8 %**	**51.0 %**
**Fatty acids**	**99.0 %**	**99.3 %**	**95.7 %**	49.7 **%**	**50.3 %**	0.4 %	0.4 %
**Alcohols**	1.4 %	1.4 %	9.3 **%**	0.8 **%**	0.1 %	0.0 %	0.0 %
**Alkadienes**	0.8 %	0.0 %	0.0 %	0.0 %	0.0 %	0.0 %	0.1 %
**Aldehydes**	0.8 %	0.2 %	0.8 %	9.3 %	1.4 %	0.2 %	8.7 %
**Keto acids**	0.0 %	0.0 %	0.0 %	0.0 %	3.7 %	0.0 %	0.0 %
**Terpenes**	1.1 %	0.0 %	4.6 %	**54.1 %**	**54.1 %**	0.0 %	0.0 %
**Terpene alcohols**	0.0 %	0.0 %	0.1 %	9.3 %	1.4 %	0.0 %	8.7 %
**Lactones**	3.7 %	0.0 %	0.0 %	0.0 %	0.0 %	0.0 %	0.0 %

In our analysis of 16 species, the Bombini (bumblebees) include the genus *Bombus*, Meliponini (stingless bees) include the genera *Melipona*, *Scaptotrigona*, *Friesella* and *Schwarziana*, Corbiculate bees correspond to all Bombini, Meliponini and *Apis mellifera* honeybees, Centris + Corbiculate bees to *Centris analis* and all Corbiculate bees, Apidae *sensu strictu* to *Amegilla dawsoni*, *Centris analis* and all Corbiculate bees, Megachile + Apidae *sensu strictu* to *Megachile rotundata* plus all Apidae *s. s.* and,Apidae *sensu lato* to all species included in our analysis. Likelihoods higher than 50 % are shown in bold

## Discussion

Overall, our results provide the first direct demonstration that many of the queen signals of Corbiculate bees are likely derived from fertility cues that were already present in solitary ancestors that lacked reproductive castes. In particular, our ancestral state reconstruction demonstrates that queen signals belonging to several distinct chemical classes, including linear alkanes, alkenes, ester and fatty acids, are likely derived from ancient fertility signals present in the common ancestor of all extant bee species, and originated at least ca. 120 Mya [36]. Similarly, we find that branched alkane and terpene queens signals are probably also derived from ancestral fertility signals, and started to be used before the origin of eusociality in Corbiculate bees, ca. 65 Mya [35, 52, 53], even though they had a more recent origin. By contrast, keto acids, such as 9-ODA, as well as aldehydes, alcohols, alkadienes, terpene alcohols and lactones are found to be used as queen or fertility signals only in very specific lineages. Overall, the diversity of fertility-linked compounds in both social and solitary bees was much higher than in other hymenopteran groups, including ants or wasps [12].

In general, our phylogenetic analysis adds credence to the idea that also in bees, compounds present on the cuticle, including both saturated and unsaturated hydrocarbons as well as other classes of compounds, such as ester and fatty acids, may act as conserved and ancient queen pheromones [12, 24]. In fact, direct evidence for cuticular compounds being used as sterility-inducing queen signals are now available for bumblebees, and strong suggestive evidence for their use has been provided also in honeybees and stingless bees. In particular, in the buff-tailed bumblebee *Bombus terrestris*, it has been shown that the linear alkane pentacosane inhibits worker ovary development [12, 21] but that some queen-characteristic esters did not suppress worker reproduction [12], even though they were still suggested to have a role in signalling fertility [20]. Similarly, in the stingless bees *Friesella schrottkyi*, queen cuticular extracts have been shown to inhibit worker ovary activation, and several hydrocarbons, including pentacosane, elicited an electroantennographic response, which suggests that cuticular compounds may act as queen pheromones in this species as well [32]. Finally, even in the well-studied honeybee, it is likely that some cuticular compounds complement the well-characterised mandibular gland compounds [54–56] in producing bio-active queen pheromone, since queens from which the mandibular gland was removed still inhibited worker reproduction [28, 29] and that extracts of the tergal glands, which produce specific compounds on the dorsal part of queen’s cuticle [30, 31], also partially inhibited worker ovary development. Specific bioactive cuticular compounds have as yet not been identified, but queen-specific N-15 long-chain alkenes [31] or specific esters or fatty acids [30], such as hexadecanoic acid, (*Z*)-9-octadecenoic acid, hexadecanoic and octadecenoic acid methyl ester or decyl decanoate [57, 58], are amongst the possibilities.

If it is correct that some groups of social insect queen pheromones were co-opted from fertility signals that were already present in solitary ancestors, the question arises what function these fertility-linked compounds had in the solitary species. Previously, three main possibilities have been suggested, namely that they originally served as sex pheromones, that they were a by-product of ovarian activation, or that they were involved in regulating egg-laying, reviewed in [24]. Although these hypotheses are not mutually exclusive, for bees, especially this first hypothesis appears to be well supported, given that in *Apis* honeybees, the major queen pheromone component 9-ODA [54, 56, 59, 60] also doubles up as a male attractant sex pheromone [61–63], and that queen tergal gland secretion has been shown to enhance the effect of 9-ODA both in terms of inhibiting worker ovary activation [64] as well as in enhancing the queen’s attractiveness to drones [65]. Moreover, in the eusocial Halictine bee *Lasioglossum malachurum*, a synthetic blend of the fertility-linked saturated lactones applied onto dummy bees was able to induce male inspection and pouncing behaviour (though not actual copulation behaviour, which appeared to be regulated by isopentenyl esters of unsaturated fatty acids) [66]. In addition, in the solitary bee *Colletes cunicularius*, several fertility-linked long-chain alkenes have also been shown to act as contact sex pheromones [67, 68] and in *Megachile rotundata,* alkene fraction extracts from virgin females, containing also the compounds linked to fertility, were shown to attract males in bioassays [69]. In all cases mentioned above, sex pheromones were characterised or presumed to be a blend of compounds functioning synergistically to attract males and elicit mating behaviour, and could contain both fertility-linked compounds as well as compounds which were produced mainly by virgin females. Reduced production of non-fertility linked sex pheromones or increased production of compounds that inhibit male copulatory behaviour could explain the loss of the attractiveness of females after mating [70, 71], although change in context [72] or transfer of anti-aphrodisiac pheromones by the males themselves during mating has also been shown to be important in some cases (e.g. the transfer of anti-aphrodisiac s fatty acid, which are transferred as part of the male mating plug in bumblebees, [73], or the direct transfer of anti-aphrodisiac male cuticular compounds during mating in *Lasioglossum zephyrum*, *L. malachurum* and *Osmia rufa* [70, 74]. Either way, it is clear that a subset of fertility-linked sex pheromones could readily be co-opted as queen pheromones after the origin of eusociality [24]. Possibly, the fact that queen and fertility signals double up as sex pheromones in several bee species may also explain the relatively large diversity found in fertility-linked compounds, as sex pheromones are generally subject to diversifying selection [75, 76].

In the future, several of the hypotheses supported by our current dataset may be further validated by looking at the degree of evolutionary conservation in the expression levels of the actual underlying enzymes involved in the biosynthesis of different fertility-linked compounds [10, 40, 43], as well as by investigating conservation at the receptor level [77, 78]. Indeed, much ongoing research is targeted towards elucidating the roles of different enzymes in synthetizing different cuticular compounds in social insects, including those of different desaturases, elongases and decarboxylases involved in hydrocarbon biosynthesis [50, 79, 80], as well as those of lipophorins, involved in hydrocarbon transport [81, 82]. Recently, the enzymes involved in the production of the honeybee QMP blend were also described [83, 84], and the olfactory receptor AmOR11 was identified as the putative receptor for 9-ODA [85]. Together with targeted bioassays to test the bioactivity of particular fertility signals, such genomic studies may in the future yield unprecedented insight into the evolution of a key signalling system in insects, and allow us to uncover how social signalling systems were built on the groundplan of solitary ancestors [86, 87].

## Conclusions

In a recent study, it was suggested that queen pheromones in different groups of social insects had been co-opted from ancient fertility signals present in common solitary ancestors [12]. Based on the analysis of fertility-linked compounds in different species of social bees and their solitary ancestors, our analysis provides direct support for this hypothesis. In particular, our results show that compounds belonging to four different chemical classes (linear alkanes, alkenes, esters and fatty acids) are used as queen signals across several species of social bees, and that these same types of compounds were also present as fertility signals in their solitary ancestors. Other classes of compounds, however, only emerged later on in the evolutionary history of bees, or are specific to particular lineages.

## Additional file

Additional file 1:
**Detailed data collection methodology, additional figure S1 with ancestral state reconstruction of the remaining six classes of chemical compounds and datasets used for the phylogenetic analysis.** (DOCX 2511 kb)
