# Supplementary material for: The origin and evolution of queen and fertility signals in Corbiculate bees
Source: BMC Evol Biol. 2015 Nov 16;15:254. doi: 10.1186/s12862-015-0509-8 (PMC4647589; doi:10.1186/s12862-015-0509-8)
Supplement: Additional file 1: — Detailed data collection methodology, additional figure S1 with ancestral state reconstruction of the remaining six classes of chemical compounds and datasets used for the phylogenetic analysis. (DOCX 2511 kb) [file 12862_2015_509_MOESM1_ESM.docx]

**Supplemental material**

*Collecting and rearing bees*

To collect *Centris analis*, five wooden blocks of trap-nests were set up at the experimental apiary of the University of São Paulo (Ribeirão Preto, SP, Brazil) containing 150 nesting cavities. A black cardboard tube of 8 cm of length and 6 mm of diameter was provided for nesting. The trap-nests were examined daily for 2 months (December 2013 - January 2014) to check for the occurrence of nesting *Centris analis* bees. Egg-laying females were collected when such individuals were seen visiting the nest cavities in the trap nests. In addition, nests constructed in the cardboard tubes were brought to the laboratory in order to collect newly emerged virgin females. Collected nests were kept at room temperature inside falcon tubes closed with a mesh and individuals were collected on the day they emerged and then anaesthetized with carbon dioxide before being freeze killed at -20^o^ C for chemical analysis. Newly emerged *C. analis* bees usually fly from the nest as soon as they emerge from their cell and present very advanced cuticular maturation when compared to social species [1]. Hence, there was no strong confounding of ovary development with cuticular maturation, although ovary development and mating status was of course unavoidably linked with age. Given that this is the natural situation, however, we did not consider this a problem for our analysis. For *Scaptotrigona depilis,* five colonies were used from the experimental apiary of the University of São Paulo of which all five queens were collected and sampled together with fifteen workers from the same colonies.

*Chemical analyses*

Bees were extracted in 1 ml of pentane (HPLC, Sigma-Aldrich) for 10 minutes, after which the samples were evaporated at room temperature to dryness and diluted in 200 µl pentane. Samples were analysed on a SHIMADZU QP 2010 ULTRA coupled gas chromatograph/mass spectrometer coupled with a DB-5ms capillary column (30 m x 0.25 mm x 0.25 µm) in which 2 µL of the pentane extract was injected. The method had an initial temperature profile consisting of 1 minute at 70 °C, two temperature ramps from 70 °C to 150 °C at 20 °C min^-1^ and from 150 °C to 320 °C at 3 °C min^-1^, after which the final temperature of 320 °C was held for 15 minutes. We used helium as a carrier gas at a flow rate of 1 mL min^-1^, splitless injection, an inlet temperature of 280 °C, and a final pressure of 75 kPa. The electron ionization voltage was auto-tuned to enhance the acquisition performance according to the molecular weight of the compounds, and the ion source temperature was set to 300 °C. Peaks in the chromatogram were integrated using GCMS Solutions software, and substances which comprised more than 0.01 % of total peak area in queens or mature females were selected for further analysis, after exclusion of any contaminants. Individuals were separated into reproductively mature and reproductively immature females for *C. analis* and queens and workers for *S. depilis* in order to assess which chemical compounds were linked with fertility. Fertility linked compounds were then ranked by their effect size which was calculated in terms of the Cohen’s *D*, i.e. the difference in mean relative abundance of the focal chemical between the two groups divided by the pooled standard deviation. Finally, compounds that were characteristic for queens (in *Scaptotrigona*) or mature egg-laying females (in *Centris*) were identified based on spectral matches with compounds in the NIST 2014 library or with synthetic standards (C14ald-C20ald, provided by Prof. Jocelyn Millar, University of California, Riverside), expected diagnostic or molecular ions in the mass spectra as well as based on retention index matches in the NIST 2014 [2] or Pherobase [3] retention index libraries (Tables S1 and S2). The retention indices of our compounds were calculated using cubic spline interpolation based on the elution times of an external C7 to C40 linear alkane ladder standard (49452-U, Supelco).

Fertility-linked chemicals in *Centris analis* and *Scaptotrigona depilis*

There was a high diversity of cuticular chemical compound classes present in both species. Among all substances present, compounds belonging to the classes of linear and branched alkanes, alkenes, aldehydes, esters, terpenes and terpene alcohols were found to be characteristic for breeding females in *C. analis* (table 1), whereas in *S. depilis* fertility-linked compounds belonging to the classes of the linear and branched alkanes, alkenes and esters were found to be characteristic for queens (table 2). Whenever a compound had specificity for either mature individuals or queens, and Cohens’ *D* was greater than 2, it was considered fertility-linked and scored as such in the matrix that we used for our ancestral state reconstruction.


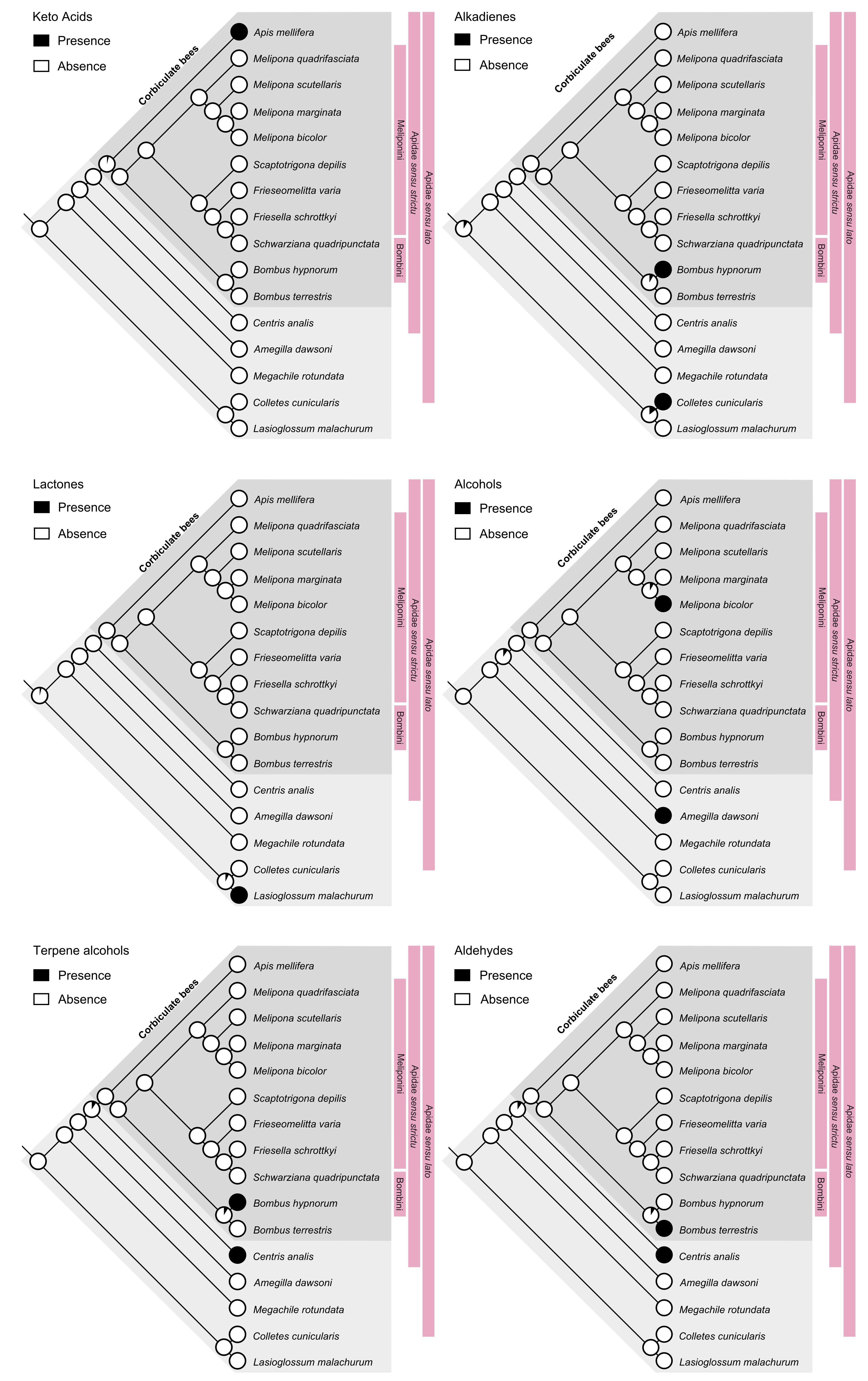


**Figure S1.** Ancestral state reconstruction of the six classes of chemical compounds which were fertility-linked only in specific lineages.

**Table S1.** Compounds showing maximum difference between reproductively mature and reproductively immature virgin females in *C. analis* measured by a Cohen’s D effect size higher than two based on whole-body pentane washes, together with the evidence that was used to identify the compounds.

| Measured RI | Library RI | Compound | Mature | | Immature | | Cohen's D | Evidence |
| --- | --- | --- | --- | --- | --- | --- | --- | --- |
|  |  |  | (n=5) | SD | (n=8) | SD |  |  |
| 1541 | 1539 | Nerolidol | 0.01 | 0.00 | 0.01 | 0.00 | 3.40 | Spectral and RI match library |
| 1823 | 1822 | Hexadecanal | 0.01 | 0.00 | 0.01 | 0.00 | 3.12 | Spectral and RI match synthetic standard |
| 2227 | 2224 | Eicosanal | 0.13 | 0.04 | 0.02 | 0.01 | 3.99 | Spectral and RI match synthetic standard |
| 2343 | 2338 | 11-MeC23 | 0.01 | 0.00 | 0.00 | 0.00 | 2.82 | Ions 168, 196 and RI match |
| 2535 | 2533 | Mix of 13- and 11-MeC25 | 0.03 | 0.01 | 0.01 | 0.00 | 2.57 | Ions 168, 141, 196, 224, 366 and RI match |
| 2546 | 2540 | 7-MeC25 | 0.95 | 0.54 | 0.11 | 0.09 | 2.53 | Ions 112, 281, 366 and RI match |
| 2762 | - | Decyl palmitoleate | 1.32 | 0.52 | 0.23 | 0.07 | 3.47 | Ions 152, 236, 394 |
| 2830 | 2847 | Squalene^a^ | 0.27 | 0.12 | 0.04 | 0.01 | 3.25 | Spectral and RI match library |
| 2977 | 2995 | Triacontene | 0.32 | 0.09 | 0.14 | 0.02 | 3.27 | Ions 83, 97, 111, 420 and RI match |
| 3000 | 3000 | Triacontane | 0.12 | 0.03 | 0.04 | 0.01 | 3.90 | Ion 422 and RI match |
| 3356 | - | Unidentified ester | 2.37 | 0.98 | 0.28 | 0.07 | 3.51 | Ions 153, 171 |

^a^ This compound is sometimes found as a contaminant present in human sweat. In our case, however, we confirmed it was not a contaminant, as it we did not handle the bees with our hands before sampling, and the compound was consistently present in the *Centris analis* samples but absent in our blanks and our *Scaptotrigona depilis* samples. Previously, the compound has also been confirmed to be present in In addition, this chemical compound in known to be present on the cuticle of several hymenopteran species including bumblebees, stingless bees and Ponerini ants [3].

**Table S2.** Compounds showing maximum difference between queens and workers in *S. depilis* measured by a Cohen’s D effect size higher than two based on whole-body pentane washes, together with the evidence that was used to identify the compounds.

| Measured RI | Library RI | Compound | Queen | | Worker | | Cohen's D | Evidence |
| --- | --- | --- | --- | --- | --- | --- | --- | --- |
|  |  |  | (n=5) | SD | (n=15) | SD |  |  |
| 1963 | - | Unidentified ester | 2.97 | 2.16 | 0.06 | 0.03 | 2.56 | Ions 82, 138, 166, 194 |
| 1975 | 1959 | Decyl octanoate | 1.07 | 0.51 | 0.04 | 0.03 | 3.32 | Ions 57, 127, 145 and RI match |
| 1984 | 1985 | Tetradecyl butyrate | 1.53 | 1.32 | 0.05 | 0.03 | 2.25 | Ions 71, 89, 168, 196, 241 and RI match |
| 2075 | 2080 | Heneicosene | 0.13 | 0.05 | 0.06 | 0.04 | 2.31 | Ions 83, 97, 111, 294 and RI match |
| 2105 | 2100 | Heneicosane | 0.13 | 0.07 | 0.04 | 0.02 | 2.64 | Ion 296 and RI match |
| 2141 | 2143 | 7-MeC21 | 0.16 | 0.06 | 0.07 | 0.03 | 2.38 | Ions 112, 224 and RI match |
| 2155 | - | Unidentified ester | 0.20 | 0.05 | 0.04 | 0.03 | 3.79 | Ions 82, 138, 166, 194 |
| 2162 | - | Unidentified ester | 1.85 | 1.70 | 0.05 | 0.03 | 2.15 | Ions 82, 138, 166, 222 |
| 2173 | 2177 | Dodecyl octanoate | 1.58 | 0.36 | 0.07 | 0.04 | 4.29 | Ions 57, 145, 168 and RI match |
| 2200 | 2200 | Docosane | 0.15 | 0.03 | 0.09 | 0.09 | 2.40 | ion 310 and RI match |
| 2276 | 2289 | Tricosene | 11.69 | 2.72 | 0.09 | 0.05 | 4.30 | Ions 83, 97, 111, 322 and RI match |
| 2301 | 2300 | Tricosane | 6.44 | 0.77 | 0.18 | 0.11 | 4.63 | Ion 324 and RI match |
| 2335 | 2338 | 11-MeC23 | 0.78 | 0.26 | 0.10 | 0.11 | 3.74 | Ions 168, 196, 338 and RI match |
| 2341 | 2341 | 7-MeC23 | 0.16 | 0.05 | 0.05 | 0.03 | 3.33 | Ions 112, 252, 338 and RI match |
| 2350 | - | Unidentified ester | 1.43 | 0.28 | 0.05 | 0.04 | 4.42 | Ions 82, 138, 166, 194 |
| 2370 | 2375 | Dodecyl decanoate | 1.00 | 0.41 | 0.05 | 0.03 | 3.53 | Ions 155, 173, 168, 340 and RI match |
| 2373 | 2375 | Tetradecyl octanoate | 0.59 | 0.19 | 0.05 | 0.03 | 3.83 | Ions 127, 145, 1196, 340 and RI match |
| 2475 | 2475 | Pentacosene | 2.39 | 0.48 | 0.18 | 0.16 | 4.32 | Ions 83, 97, 111, 350 and RI match |
| 2534 | 2534 | Mix of 13- and 11-MeC25 | 0.49 | 0.11 | 0.17 | 0.09 | 2.74 | Ions 168, 196, 224 and RI match |
| 2540 | 2540 | 7-MeC25 | 0.34 | 0.19 | 0.08 | 0.03 | 2.63 | Ions 112, 280 and RI match |
| 2549 | 2553 | Mix of 5- and 3- MeC25 | 0.52 | 0.12 | 0.15 | 0.05 | 3.73 | Ions 85, 309, 337 and RI match |
| 2567 | 2574 | Dodecyl dodecanoate | 0.53 | 0.47 | 0.05 | 0.04 | 2.12 | Ions 168, 183, 201, 368 and RI match |
| 2600 | 2600 | Hexacosane | 0.70 | 0.23 | 0.28 | 0.12 | 2.42 | ion 366 and RI match |
| 2773 | 2772 | 3-MeC27 | 0.54 | 0.13 | 0.28 | 0.11 | 2.05 | Ions 57, 337, 365 and RI match |
| 2943 | - | Decyl oleate | 0.72 | 0.61 | 0.10 | 0.06 | 2.12 | Ions 152, 180, 222, 264, 422 |
| 3100 | 3100 | Hentriacontane | 1.58 | 0.80 | 0.25 | 0.11 | 2.93 | Ion 436 and RI match |
| 3299 | 3300 | Tritriacontane | 0.39 | 0.17 | 0.13 | 0.07 | 2.64 | Ion 464 and RI match |
| 4487 | - | Tetracosyl oleate | 1.99 | 0.94 | 0.39 | 0.21 | 2.97 | Ion 97, 180, 222, 264 |

**Table S3.** Fertility-linked chemical compounds across 16 species of social and solitary bees.

| **Species** | **Chemical Compounds** | **References** |
| --- | --- | --- |
| ***Apis mellifera*** | C23:1; C25:1; C27:1; C29:1; C31:1; C33:1; C35:1; C37:1; C31:2; C33:2; C35:2; C37:2\| Palmitic acid; 9-Stearic acid \| ethyl palmitate; methyl palmitate; methyl linoleate; Tetradecyl dodecanoate; Tetradecyl tetradecanoate; Tetradecyl-(Z)-9-hexadecenoate; Tetradecyl hexadecanoate; Hexadecyl tetradecanoate; Hexadecenyl hexadecanoate; Tetradecyl-(Z)-9-octadecenoate; Tetradecyl-(Z)-9-hexadecenoate; Hexadecyl hexadecanoate; Octadecyl hexadecanoate \| 9-ODA, 9-HDA \| β-ocimene | [4-20] |
| ***Melipona quadrifasciata*** | n-C24; n-C26; n-C28 \| 9-MeC27; 7-MeC27; MeC29; MeC31 \| C26:1; C27:1; C28:1; C29:1; C30:1; C31:1 | [21] |
| ***Melipona scutellaris*** | n-C19; n-C21; n-C22; n-C23; n-C25 \| Stearyl acetate; Arachidyl acetate; Ethyl oleate | [22] |
| ***Melipona marginata*** | n-C27; n-C28; n-C29; n-C31 \| 5-MeC25 \| C25:1 \| C27:2 | [23] |
| ***Melipona bicolor*** | n-C26; n-C28; n-C30; n-C32 \| 5;11+5;19+11;13-diMeC25; 11;13;15-triMeC27; 5-MeC27; 10+14+15-MeC28; 11;13;15-triMeC29; 5+15-MeC29 \| C26:1; C31:1 \| Dodecanol | [24] |
| ***Scaptotrigona depilis*** | n-C21; n-C22; n-C23; n-C26; n-C31; n-C33 \| 7-MeC21; 11-MeC23; 7-MeC23; 7-MeC25; Mix of 13-;11-MeC25; Mix of 5-,3-MeC25; 3-MeC27 \| C23:1; C25:1 \| Decyl octanoate; Tetradecyl butyrate; Dodecyl octanoate; Dodecyl decanoate; Tetradecyl octanoate; Dodecyl dodecanoate; Decyl oleate; Tetracosyl oleate | This study |
| ***Frieseomelitta varia*** | 7+9+14-C31:1; 9;17+9;19-C31:2; 8;22-C31:2; 5+7+12-C29:1; 9;17+7;17-C29:2; 5+7+10+13-C27:1 \| ethyl oleate | [25] |
| ***Friesella schrottkyi*** | n-C21; n-C23; n-C25; n-C26; n-C27; n-C29 \| 11+5-MeC25; 11-MeC27; 11;15+5;17-diMeC27; 11-MeC28; 11+13-MeC29; 13;15+5;19-diMeC29 | [26, 27] |
| ***Schawarziana quadripunctata*** | n-C26; n-C27 | [28] |
| ***Bombus hypnorum*** | Me-C23; Me-C25 \| 11-C25:1; 7-C25:1n \| C27:2; C29:2; C30:2 \| geranyl citronellol | [29] |
| ***Bombus terrestris*** | n-C21; n-C23; n-C25; n-C27; n-C29\| 9+11-MeC21\| C23:1; C25:1; C27:1; C29:1; C29:2; C31:1 \| decyl tetradecanoate; dodecyl dodecanoate; dodecyl hexadecenoate; dodecyl octadecenoate; eicosyl oleate; docosyl oleate; tetracosyl oleate; hexacosyl oleate \| C30-Ald; C32-Ald | [30-36] |
| ***Centris analis**** | n-C30 \| 11-MeC23; Mix of 13-,11-MeC25; 7-MeC25 \| C16-Ald; C20-Ald \| Squalene \| Nerolidol \| Decyl palmitoleate | This study |
| ***Amegilla dawsoni**** | n-C23; n-C24 \| C25:1 \| Tetramethyl-hexadecatrienol \| Myristic acid; Palmitic acid | [37] |
| ***Megachile rotundata**** | n-C21; n-C22 \| 13+11-MeC35; 15+13-MeC31 \| 5-C23:1; 5-C24:1; 9-C24:1; 5-C25:1; 5-C26:1; 13-C26:1; 11-C26:1; 11-C27:1; 13-C27:1; 9-C27:1; 5-C27:1; 13-C29:1; 11-C29:1; 9-C29:1; 5-C29:1; 11-C31:1; 9-C31:1 ; 7-C31:1; 5-C31:1 \| Myristic acid; Linoleic acid | [38] |
| ***Colletes cunicularis**** | n-C21; n-C23; n-C25 \| 9-C23; 7-C23; 9-C24; 9-C25; 7-C25; 11-C29; 9-C29; 9-C31 \| Oleic acid; Linoleic acid \| Dodecyl tetradecanoate | [39] |
| ***Lasioglossum malachurum**** | n-C21; n-C23; n-C27; n-C29 \| 7+9-C23:1; 7+9-C25:1; 7+9-C27:1 \| ethyl eicosenoate \| docosenoic acid; tetracosanoic acid \| 20-Eicosanolide; 22-Docosanolide; 24-Tetracosanolide | [40] |

* Based on the cuticular chemical profile of mated vs. virgin females.

**Table S4.** Data matrix specifying the presence of fertility-linked compounds belonging to particular biosynthetic groups of compounds, as used in our maximum likelihood ancestral state reconstruction.

|  | **Linear alkane** | **Branched alkane** | **Alkene** | **Alkadiene** | **Aldehyde** | **Alcohol** | **Fatty acid** | **Keto acid** | **Ester** | **Terpene** | **Terpene alcohol** | **Lactones** |
| --- | --- | --- | --- | --- | --- | --- | --- | --- | --- | --- | --- | --- |
| *C. cunicularis* | 1 | 0 | 0 | 1 | 0 | 0 | 1 | 0 | 1 | 0 | 0 | 0 |
| *L. malachurum* | 1 | 0 | 1 | 0 | 0 | 0 | 1 | 0 | 1 | 0 | 0 | 1 |
| *A. dawsoni* | 1 | 1 | 0 | 0 | 0 | 1 | 1 | 0 | 0 | 0 | 0 | 0 |
| *M. otundata* | 1 | 1 | 1 | 0 | 0 | 0 | 1 | 0 | 0 | 0 | 0 | 0 |
| *C. analis* | 1 | 1 | 1 | 0 | 1 | 0 | 0 | 0 | 1 | 1 | 1 | 0 |
| *A. mellifera* | 0 | 0 | 1 | 0 | 0 | 0 | 1 | 1 | 1 | 1 | 0 | 0 |
| *B. terrestris* | 1 | 1 | 1 | 0 | 1 | 0 | 0 | 0 | 1 | 0 | 0 | 0 |
| *B. hypnorum* | 0 | 1 | 1 | 1 | 0 | 0 | 0 | 0 | 0 | 0 | 1 | 0 |
| *F. schrottkyi* | 1 | 1 | 0 | 0 | 0 | 0 | 0 | 0 | 0 | 0 | 0 | 0 |
| *S. quadripunctata* | 1 | 0 | 0 | 0 | 0 | 0 | 0 | 0 | 0 | 0 | 0 | 0 |
| *M. scutellaris* | 1 | 0 | 0 | 0 | 0 | 0 | 0 | 0 | 1 | 0 | 0 | 0 |
| *M. bicolor* | 1 | 1 | 1 | 0 | 0 | 1 | 0 | 0 | 0 | 0 | 0 | 0 |
| *M. marginata* | 1 | 1 | 1 | 0 | 0 | 0 | 0 | 0 | 0 | 0 | 0 | 0 |
| *M. quadrifasciata* | 1 | 1 | 1 | 0 | 0 | 0 | 0 | 0 | 0 | 0 | 0 | 0 |
| *F. varia* | 0 | 0 | 1 | 0 | 0 | 0 | 0 | 0 | 1 | 0 | 0 | 0 |
| *S. depilis* | 1 | 1 | 1 | 0 | 0 | 0 | 0 | 0 | 1 | 0 | 0 | 0 |

**References**

1. Elias-Neto M, Nascimento AL, Bonetti AM, Nascimento FS, Mateus S, Garófalo CA, Bitondi MM: Heterochrony of cuticular differentiation in eusocial corbiculate bees. *Apidologie* 2014, 45(4):397-408.

2. Linstrom P, Mirokhin Y, Tchekhovskoi D, Yang X: NIST Standard Reference Database. In*.*, 2.2g edn; 2014.

3. El-Sayed A: The pherobase: database of pheromones and semiochemicals. 2012.

4. Voogd S: The influence of a queen on the ovary development in worker bees. *Experientia* 1956, 12(5):199-201.

5. Butler C, Fairey EM: The role of the queen in preventing oogenesis in worker honeybees. *J Apic Res* 1963, 2(1):14-18.

6. Plettner E, Otis G, Wimalaratne P, Winston M, Slessor K, Pankiw T, Punchihewa P: Species-and caste-determined mandibular gland signals in honeybees (*Apis*). *J Chem Ecol* 1997, 23(2):363-377.

7. Hoover SER, Keeling CI, Winston ML, Slessor KN: The effect of queen pheromones on worker honey bee ovary development. *Naturwissenschaften* 2003, 90(10):477-480.

8. Tan K, Yang M, Radloff S, Pirk CWW, Crewe RM, Phiancharoen M, Hepburn R, Oldroyd BP: Worker reproduction in mixed-species colonies of honey bees. *Behav Ecol* 2009, 20(5):1106-1110.

9. Smith RK, Taylor Jr OR: Unsaturated extracted hydrocarbon caste differences between European queen and worker honey bees, *Apis mellifera* L. (Hymenoptera: Apidae). *J Kans Entomol Soc* 1990:369-374.

10. Wossler TC, Crewe RM: Mass spectral identification of the tergal gland secretions of female castes of two African honey bee races (*Apis mellifera*). *J Apic Res* 1999, 38(3-4):137-148.

11. Wossler TC, Crewe RM: Honeybee queen tergal gland secretion affects ovarian development in caged workers. *Apidologie* 1999, 30(4):311-320.

12. Gilley D, DeGrandi-Hoffman G, Hooper J: Volatile compounds emitted by live European honey bee (*Apis mellifera* L.) queens. *J Insect Physiol* 2006, 52(5):520-527.

13. Maisonnasse A, Lenoir JC, Beslay D, Crauser D, Le Conte Y: E-β-ocimene, a volatile brood pheromone involved in social regulation in the honey bee colony (*Apis mellifera*). *PLoS One* 2010, 5(10):e13531.

14. Maisonnasse A, Lenoir JC, Costagliola G, Beslay D, Choteau F, Crauser D, Becard J-M, Plettner E, Le Conte Y: A scientific note on E-β-ocimene, a new volatile primer pheromone that inhibits worker ovary development in honey bees. *Apidologie* 2009, 40(5):562-564.

15. Mohammedi A, Paris A, Crauser D, Le Conte Y: Effect of aliphatic esters on ovary development of queenless bees (*Apis mellifera* L.). *Naturwissenschaften* 1998, 85(9):455-458.

16. Keeling CI, Slessor KN, Higo HA, Winston ML: New components of the honey bee (*Apis mellifera* L.) queen retinue pheromone. *Proc Natl Acad Sci USA* 2003, 100(8):4486-4491.

17. Slessor KN, Winston ML, Le Conte Y: Pheromone communication in the honeybee (*Apis mellifera* L.). *J Chem Ecol* 2005, 31(11):2731-2745.

18. Le Conte Y, Bécard JM, Costagliola G, de Vaublanc G, El Maâtaoui M, Crauser D, Plettner E, Slessor KN: Larval salivary glands are a source of primer and releaser pheromone in honey bee (*Apis mellifera* L.). *Naturwissenschaften* 2006, 93(5):237-241.

19. Hoover SE, Winston ML, Oldroyd BP: Retinue attraction and ovary activation: responses of wild type and anarchistic honey bees (*Apis mellifera*) to queen and brood pheromones. *Behav Ecol Sociobiol* 2005, 59(2):278-284.

20. Katzav-Gozansky T, Soroker V, Francke W, Hefetz A: Honeybee egg-laying workers mimic a queen signal. *Ins soc* 2003, 50(1):20-23.

21. Borges AA, Ferreira-Caliman MJ, Nascimento FS: Characterization of cuticular hydrocarbons of diploid and haploid males, workers and queens of the stingless bee *Melipona quadrifasciata*. *Ins soc* 2012.

22. Kerr WE, Jungnickel H, Morgan ED: Workers of the stingless bee *Melipona scutellaris* are more similar to males than to queens in their cuticular compounds. *Apidologie* 2004, 35(6):611-618.

23. Falcón T, Mateus S, Zucchi R, Nascimento FS: Chemical identity of recently emerged workers, males, and queens in the stingless bee *Melipona marginata*. *Apidologie* 2013.

24. Abdalla FC, Jones GR, Morgan ED, Cruz-Landim Cd: Comparative study of the cuticular hydrocarbon composition of *Melipona bicolor* Lepeletier, 1836 (Hymenoptera, Meliponini) workers and queens. *Gen Mol Res* 2003:191-199.

25. Nunes TM, Turatti ICC, Lopes NP, Zucchi R: Chemical Signals in the Stingless Bee, *Frieseomelitta varia*, Indicate Caste, Gender, Age, and Reproductive Status. *J Chem Ecol* 2009, 35(10):1172-1180.

26. Nunes TM, Morgan ED, Drijfhout FP, Zucchi R: Caste-specific cuticular lipids in the stingless bee *Friesella schrottkyi*. *Apidologie* 2010, 41(5):579-588.

27. TM N: Sobre a ecologia química de Friesella schrottkyi Friese 1900 (Hymenoptera, Apidae, Meliponini) com ênfase na regulação social. Ribeirão Preto: University of Sao Paulo; 2012.

28. Nunes T, Turatti I, Mateus S, Nascimento F, Lopes N, Zucchi R: Cuticular hydrocarbons in the stingless bee *Schwarziana quadripunctata* (Hymenoptera, Apidae, Meliponini): differences between colonies, castes and age. *Gen Mol Res* 2009, 8:589-595.

29. Ayasse M, Marlovits T, Tengoe J, Taghizadeh T, Francke W: Are there pheromonal dominance signals in the bumblebee *Bombus hypnorum* L (Hymenoptera, Apidae)? *Apidologie* 1995, 26:163-180.

30. Hefetz A, Taghizadehr T, Francke W: The exocrinology of the queen bumble bee *Bombus terrestris* (Hymenoptera: Apidae, Bombini). *Zeitschrift für Naturforschung C* 1996, 51(5-6):409-422.

31. Cahlíková L, Hovorka O, Ptáček V, Valterová I: Exocrine gland secretions of virgin queens of five bumblebee species (Hymenoptera: Apidae, Bombini). *Zeitschrift für Naturforschung C* 2004, 59(7-8):582-589.

32. Sramkova A, Schulz C, Twele R, Francke W, Ayasse M: Fertility signals in the bumblebee *Bombus terrestris* (Hymenoptera: Apidae ). *Naturwissenschaften* 2008, 95(6):515-522.

33. Van Honk CGJ, Velthuis HHW, RÖSeler PF, Malotaux ME: The mandibular glands of *Bombus terrestris* queens as a source of queen pheromones. *Entomol Exp Appl* 1980, 28(2):191-198.

34. Röseler P-F, Röseler I, Van Honk C: Evidence for inhibition of corpora allata activity in workers of *Bombus terrestris* by a pheromone from the queen's mandibular glands. *Experientia* 1981, 37(4):348-351.

35. Bloch G, Hefetz A: Reevaluation of the Role of Mandibular Glands in Regulation of Reproduction in Bumblebee Colonies. *J Chem Ecol* 1999, 25(4):881-896.

36. Van Oystaeyen A, Oliveira RC, Holman L, van Zweden JS, Romero C, Oi CA, d'Ettorre P, Khalesi M, Billen J, Wackers F *et al*: Conserved class of queen pheromones stops social insect workers from reproducing. *Science* 2014, 343(6168):287-290.

37. Simmons LW, Alcock J, Reeder A: The role of cuticular hydrocarbons in male attraction and repulsion by female Dawson's burrowing bee, *Amegilla dawsoni*. *Anim Behav* 2003.

38. Paulmier I, Bagnères A-G, Afonso CM, Dusticier G, Rivière G, Clément J-L: Alkenes as a sexual pheromone in the alfalfa leaf-cutter bee *Megachile rotundata*. *J Chem Ecol* 1999, 25(3):471-490.

39. Mant J, Brändli C, Vereecken NJ, Schulz CM, Francke W, Schiestl FP: Cuticular hydrocarbons as sex pheromone of the bee *Colletes cunicularius* and the key to its mimicry by the sexually deceptive orchid, *Ophrys exaltata*. *J Chem Ecol* 2005, 31(8):1765-1787.

40. Ayasse M, Engels W, Lubke G, Taghizadeh T, Francke W: Mating expenditures reduced via female sex pheromone modulation in the primitively eusocial halictine bee, *Lasioglossum* (*Evylaeus*) *malachurum* (Hymenoptera : Halictidae). *Behav Ecol Sociobiol* 1999, 45(2):95-106.
